# Interaction between the Stress Phase Angle (SPA) and the Oscillatory Shear Index (OSI) Affects Endothelial Cell Gene Expression

**DOI:** 10.1371/journal.pone.0166569

**Published:** 2016-11-15

**Authors:** Ronny Amaya, Limary M. Cancel, John M. Tarbell

**Affiliations:** Department of Biomedical Engineering, The City College of New York, New York, New York, United States of America; University of California Berkeley, UNITED STATES

## Abstract

Hemodynamic forces play an important role in the non-uniform distribution of atherosclerotic lesions. Endothelial cells are exposed simultaneously to fluid wall shear stress (WSS) and solid circumferential stress (CS). Due to variations in impedance (global factors) and geometric complexities (local factors) in the arterial circulation a time lag arises between these two forces that can be characterized by the temporal phase angle between CS and WSS (stress phase angle–SPA). Asynchronous flows (SPA close to -180°) that are most prominent in coronary arteries have been associated with localization of atherosclerosis. Reversing oscillatory flows characterized by an oscillatory shear index (OSI) that is great than zero are also associated with atherosclerosis localization. In this study we examined the relationship between asynchronous flows and reversing flows in altering the expression of 37 genes relevant to atherosclerosis development. In the case of reversing oscillatory flow, we observed that the asynchronous condition upregulated 8 genes compared to synchronous hemodynamics, most of them proatherogenic. Upregulation of the pro-inflammatory transcription factor NFκB p65 was confirmed by western blot, and nuclear translocation of NFκB p65 was confirmed by immunofluorescence staining. A comparative study between non-reversing flow and reversing flow found that in the case of synchronous hemodynamics, reversing flow altered the expression of 11 genes, while in the case of asynchronous hemodynamics, reversing flow altered the expression of 17 genes. Reversing flow significantly upregulated protein expression of NFκB p65 for both synchronous and asynchronous conditions. Nuclear translocation of NFκB p65 was confirmed for synchronous and asynchronous conditions in the presence of flow reversal. These data suggest that asynchronous hemodynamics and reversing flow can elicit proatherogenic responses in endothelial cells compared to synchronous hemodynamics without shear stress reversal, indicating that SPA as well as reversal flow (OSI) are important parameters characterizing arterial susceptibility to disease.

## Introduction

Hemodynamic forces play a key role in the localization of atherosclerosis. Endothelial cells (ECs) lining the blood vessels are exposed simultaneously to wall shear stress (WSS) caused by pulsatile blood flow and circumferential stress/strain (CS) caused by the distension of the wall diameter in response to the pressure pulse. Early studies by Caro et al., proposed that regions in the circulation exposed to low WSS (<4 dyne/cm^2^) are prone to develop atherosclerosis while regions with high WSS are spared from disease [1, 2]. The introduction of laser Doppler and ultrasound velocimetry facilitated more precise measurements of local hemodynamics and the characterization of flow reversal. The oscillatory shear index (OSI) was introduced to characterize the degree of shear reversal in a pulsatile flow–ranging from 0.0 in a uni-directional flow to 0.5 in a reversing flow with no mean shear direction [3]. Advances in computational fluid dynamics (CFD) allowed more precise calculations of the distribution of WSS and CS in atherogenic arterial geometries. Qiu and Tarbell showed that due to distal impedance in the circulation (global factors) and the non-linear inertia of blood flow in complex geometries (local factors), a time (phase) lag arises between the pulsatile WSS and CS. This phase lag was characterized by the temporal phase angle between CS and WSS (φ[CS]– φ[WSS]) that has been called the “Stress Phase Angle” (SPA) [4, 5].

Previous studies of the role of hemodynamic parameters in the onset of atherosclerosis correlated mean WSS and oscillatory shear index (OSI) with intimal thickening and inflammation with emphasis on the carotid bifurcation and branches of the aorta. However the general relationship of low mean WSS and high OSI with intimal thickening failed in several regions of the arterial circulation including coronary arteries that are the most diseased vessels in the circulation [6] and mildly diseased arteries [7], suggesting that hemodynamic parameters other than mean WSS or OSI may play an important role in the localization and onset of atherosclerosis.

Our lab showed in previous studies that regions in the circulation with highly asynchronous hemodynamics (SPA close to -180°) often co-localized with regions of low mean WSS and high OSI, for example in the carotid bifurcation [8] and end to end anastomoses [4]. The coronary arteries are an exception because they may have highly asynchronous SPA without low mean WSS or high OSI [9]. In addition, we showed that EC in vitro exposed to a combination of pulsatile WSS and CS without reversal (OSI = 0.0) are affected significantly by changes in SPA. EC exposed to SPA = -180° elicit a proatherogenic response inducing an atherogenic gene expression profile and the activation of the pro-inflammatory transcriptional factor NFκB[10].

In the present in vitro study of SPA effects on gene expression, we introduce a component of reversal flow equivalent to OSI = 0.15 to the synchronous and asynchronous hemodynamics and attempt to evaluate the significance of SPA variations under reversing flow. Our results show that changes in SPA can have significant effects on gene expression under reversing flow conditions, but also indicate that OSI has larger effects than SPA for many genes.

## Materials and Methods

In our previous study of the effect of SPA without flow reversal (OSI = 0) [10], we imposed sinusoidal WSS = 10 ± 10 dyn/cm^2^ and sinusoidal CS = 4 ± 4% for 7 h, and examined the same PCR array of genes as in the present study. All results were normalized by static control values. In the present study, the same CS was imposed, but a reversal component was added to WSS (WSS = 5 ± 10 dyn/cm^2^) resulting in OSI = 0.15. Loading was again applied for 7h and gene expression results were normalized by static control values.

### Characteristics of the Hemodynamic Simulator

To investigate the role of the SPA parameter in hemodynamics, it was necessary to develop a new device capable of controlling pulsatile wall shear stress (WSS) and cyclic strain (CS) over a wide range of physiological conditions. The device was thoroughly described in Amaya et al. [10]. Briefly, the device combines a customized pulsatile flow valve that is mechanically linked to the membrane stretching mechanism, and a parallel-flow chamber. Hydrostatic pressure drives flow into the pulsatile flow valve that imposes an oscillatory (nearly sinusoidal) component on the steady flow that results in a range of pulsatile conditions with the capability of controlling the mean, amplitude and frequency of WSS. The fluid flow is driven into a parallel plate flow chamber that has a flexible upper wall substrate where EC are seeded on a delimited region facing the pulsatile flow media. The mechanical motion in the device is driven by a DC motor and the mechanical forces are transmitted simultaneously to both the pulsatile valve and the upper wall stretching actuator through a mechanical linkage. A phase angle-link station that connects the motor with the pulsatile valve allows different configurations in the opening of the valve, generating any desired phase angle between the pulsatile flow and the stretching actuator. This is how the SPA is controlled while WSS and CS waveforms are maintained constant. The device is capable of applying different rates of sinusoidal WSS with mean WSS and WSS amplitude in the range (0–13 dyn/cm^2^) and the tensile cyclic strain in the range 0% - 20%. The frequency range is (0 to 2 Hz).

### Cell culture and experimental conditions

Cell culture components obtained from Sigma (St. Louis, MO) include: bovine serum albumin (BSA, 30% solution), trypsin, penicillin streptomycin, MEM (phenol red free), sodium bicarbonate, fibronectin, fetal bovine serum (FBS), L-Glutamine. Dulbecco's PBS (1x without Ca2+ and Mg2+) was from Fisher Scientific (Houston, TX). BAECs were purchased from VEC Technologies (Rensselaer, NY) and grown in T-75 flasks with 10% FBS-MEM. Silicone sheets (Down Corning Corporation, MI) were used as the substrates for cell culture. BAECs were seeded onto the upper surface of silicone membranes of 0.020" thickness. First the membranes were laser cut to the geometrical specifications of the bioreactor, and then silicone substrates were washed for 20min with a gentle detergent and autoclaved for 30 minutes. A region delimited by an area of 1.5 x 4.5 cm on each substrate was coated using bovine plasma fibronectin (30μg/ml in MEM) for 1 hour at room temperature. The BAECs between passages 3 and 7 were plated at a density of 1.0x10^5^ cells/cm^2^ onto the treated silicone substrates. ECs were grown using 10% FBS until confluency in a controlled environment (37C and 5% CO2 air). The EC monolayer reached confluency within four days.

### Experimental Design and Operating Conditions

To explore how endothelial cells acquire different phenotypes in response to vascular pulsatile WSS and CS at SPA characteristic of athero-protective and athero-prone regions, we used the hemodynamic simulator to reproduce the same sinusoidal WSS and CS waveforms, but at different phase angles. Changes in the expression of 42 genes (including housekeeping genes) after 7 hours of mechanical stimulation, were studied. This time point was selected for comparison because EC are considered nearly flow-adapted with respect to gene expression after this time exposure [11]. Details of the imposed WSS and CS conditions are given below.

**Oscillatory flow with synchronous SPA and reversal:** oscillatory flow with WSS = 5±10 dyn/cm^2^, CS = 4 ± 4%, frequency = 1 Hz, OSI = 0.15, SPA = 0°.

**Oscillatory flow with asynchronous SPA and reversal:** oscillatory flow with WSS = 5±10 dyn/cm^2^, CS = 4 ± 4%, frequency = 1 Hz, OSI = 0.15, SPA = -180°.

**Static control:** WSS = 0 dyn/cm^2^, CS = 0%.

### Fluorescence immunostaining

A specific antibody for NFkB p65 (Santacruz, California) was visualized using fluorescence microscopy. Cell-laden silicone membranes were washed twice with PBS and fixed in 1% PFA for 10 min, cut into sections, and permeabilized with Triton X-100 in PBS for 10 min. After permeabilization samples were blocked in 10% BSA and 0.1% Triton X-100 in PBS for 1h. Then the silicone sheets were incubated with polyclonal rabbit anti- NFkB primary antibodies overnight at 4°C followed by washing with 0.1% Triton X-100 in PBS. Samples were subjected to secondary antibody Alexa Fluor 488 donkey anti-goat (1:100 to 400; Santa Cruz) for 1.5h for NFκB. Samples were washed again with 0.1% Triton X-100 in PBS and mounted with vectashield mounting media with DAPI on glass slides with cover slips in contact with cells. These slides were imaged using a Nikon Eclipse TE2000-E inverted fluorescence microscope with a Photometrics Cascade 650 camera (Roper Scientific) and MetaVue 6.2r2 imaging software (Universal Imaging).

### Western blot analysis

The monolayers were washed once with ice-cold PBS and scraped from the silicone membranes with a plastic scraper in the presence of RIPA extraction buffer (1 mM NaHCO3, 2 mM PMSF, 1 mM Na3VO4, 5 mM EDTA, 10% protease and phosphatase inhibitor cocktail tablet and 1% Triton-X) followed by 30 s sonication on ice. Protein concentration was determined with the Protein determination kit from Cayman chemical (Ann Harbor, Mi) using the spectrophotometer Synergy HT from Biotek. Western blotting was carried out by standard techniques, loading 30 μg of protein into gradient precast-gels from Biorad (Berkeley, Ca) and incubating overnight with antibodies to NFκB (p65 dilution 1:1000), and the constitutively expressed protein β-actin from Cell Signalling Technologies (Beverly, MA), followed by specific secondary HRP conjugated anti-rabbit, anti-mouse and anti-goat IgG from Cell Signaling Technologies (Beverly, MA). The blots were scanned with the Biorad western blot scanner and quantified with Image J software.

### Gene expression

At the end of the stretch and shear experiments, RNA was isolated using TRIzol reagent (Invitrogen) following the manufacture’s protocol. RNA was purified using the RNeasy kit (Invitrogen), and then purified RNA was converted to cDNA by reverse transcription (RT). For analyzing gene expression, quantitative real-time PCR (RT-qPCR) was performed for 42 genes (Table 1) on the ABI PRISM 7000 sequence detection system (Applied Biosystems). Using the geNorm algorithm, βactin, B2M, and HPRT were determined to be the best performing housekeeping genes (HKG) and the geometric mean quantities of the HKG were used as the normalization factor [10]. The PCR reactions were performed in 25-μl reaction mixture volumes containing SYBR Green PCR Master Mix (Applied Biosystems), primer pairs, and cDNA. The programs for RT-qPCR were set to 15 min at 95°C, followed by 40 cycles of 17 s at 95°C, 30 s at 54°C, and 30 s at 72°C. Gene expression was calculated using the DDCt method. Following each PCR, dissociation curve analysis was used to evaluate the specificity of product amplification.

**Table 1:** Atherosclerosis Related Gene List (42 genes) for PCR Array.

**Vasoactive genes**
eNOS, EDN1(ET-1), PTGS2(COX-2)
**Tight Junctions and Adhesion Molecules**
***Tight junction*:** Occludin-1 (OCLN), zonula occludens 1 (ZO-1), VE-cadherin (CDH5)
***Cell-cell adhesion*:** ICAM1, SELE (E-Selectin), VCAM1
**Blood Coagulation**
THBD (Thrombomodulin), endothelial protein C receptor (EPCR), CD36, syndecan-1(SCD-1)
**Lipid Metabolism**
ABCA1 (ABC-1), APOE, OLR1 (oxLDL or LOX-1), ADFP (adipophilin), SCARB1 (SR BI)
**Nuclear Receptors and Inflammatory**
***Nuclear receptor*:** NR1H3 (LXRA), PPARG
***Inflammatory response*:** CCL2, CCL5 (Rantes), NFΚB1, IL6, IL8, CD40 (TNFRSF5)
**Oxidative stress**
SOD1, SOD2
**Apoptosis**
BAX, BCL2, TNFAIP3 (A20), TNFRSF6 (Fas)
**Other Genes**
ANGPT-2, PRDX2, KLF2, BMP4, GPC1, TGFB1, VEGF
**Housekeeping Genes**
HPRT, B2M, βactin

### Data analysis

Results are presented as mean ± SEM obtained from at least eleven independent experiments for gene expression. Samples were obtained from monolayers that did not show damage or desquamation. To compare the effects of SPA for fixed OSI, statistical analyses for both gene expression and Western blots were performed by two-tailed Student's t-test. To compare the effects of OSI for different SPA, statistical analysis of gene expression was performed by two-way ANOVA with the Sidak method to correct for multiple comparisons. Differences were considered significant if p<0.1 as in [10], and as suggested in a recent review of statistical inference testing [12]. We also compared results using the more stringent p<0.05. GraphPad Prism was used to perform statistical analyses.

## Results

### Effects of SPA in the Presence of Reversal Flow

**Fig 1:**
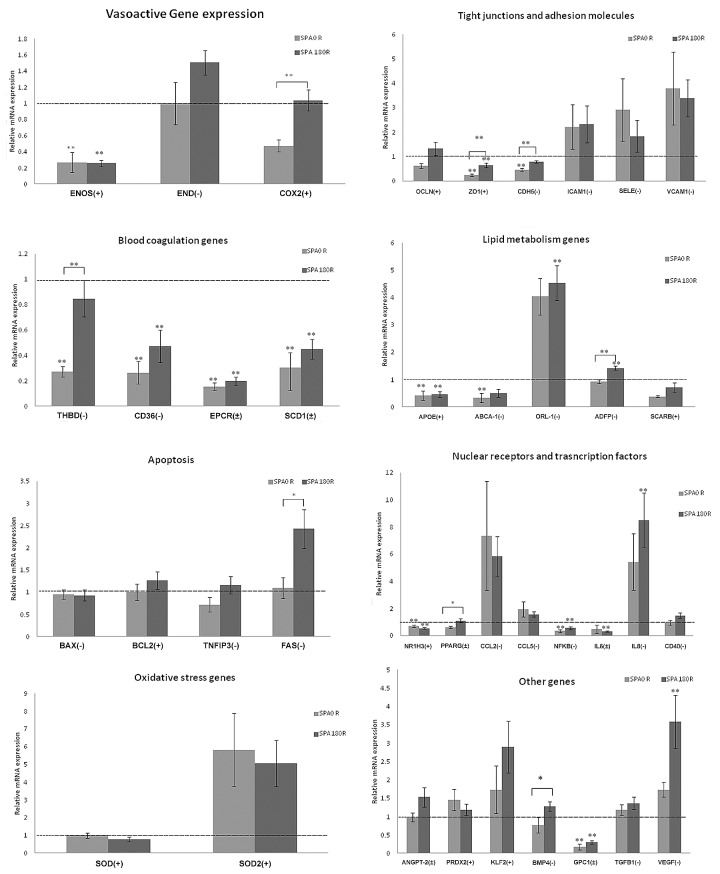
Asynchronous hemodynamics with a reversal component (OSI = 0.15) affect gene expression. In the panels above, significant differences between SPA0 R and SPA180 R are denoted by an overbar. Other significance indicators are with respect to the static control that is indicated by a dashed line at 1. * P < 0.1, ** P < 0.05 (n = 11 for SPA = -180R and n = 6 for SPA = 0R). The (+), (-) and (±) indicate anti-atherogenic, pro-atherogenic and indeterminate, respectively.

In our previous study we compared the effects of SPA without reversal flow [10] on expression of 42 genes. In this section we present analogous results for the same array of genes when there is reversal flow (OSI = 0.15). All of the results are displayed in Fig 1. A total of 8 genes (p<0.1) (5 genes p<0.05) were significantly up-regulated by the SPA = -180° condition compared to the SPA = 0° condition in the presence of flow reversal (OSI = 0.15) as summarized in Table 2. No genes were down-regulated by SPA = -180°. In our previous study of the same genes without flow reversal (OSI = 0.0), 17 genes were up-regulated by SPA = - 180° [10].

**Table 2:** Endothelial genes up-regulated by the athero-prone condition SPA = -180R relative to SPA = 0R.

Gene bank	Gene name	SPA 180R/SPA 0R	Characteristic	p- Value
AF004944	COX2	2.20	Indeterminate	0.0137
AJ313183.1	ZO1	2.71	Antiatherogenic	0.0118
XM_002694894	CDH5	1.70	Proatherogenic	0.0034
NM_001166522.1	THBD	3.10	Proatherogenic	0.0184
BC102211	ADFP	1.50	Proatherogenic	0.0006
NM_174662	FAS	2.20	Proatherogenic	0.0908
NM_181024	PPARG	1.80	Indeterminate	0.0741
BC105344	BMP4	1.70	Proatherogenic	0.06757

The third column is the ratio of gene expression the two conditions.

### Effects of OSI for SPA = 0

**Fig 2:**
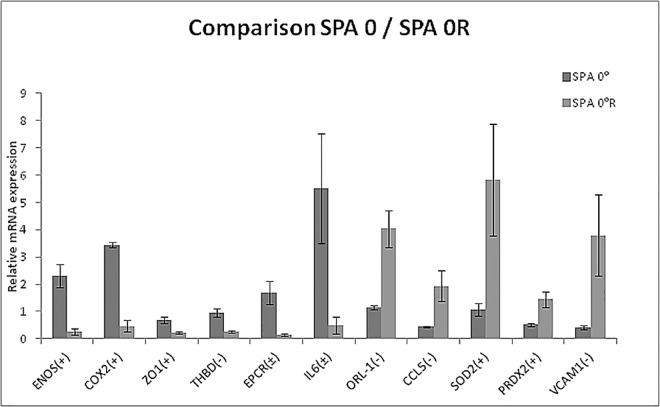
Comparative study of SPA0 vs SPA0R. All of the genes that are significantly altered (p<0.1) by reversal flow under the synchronous condition (SPA0) are shown in this graph. The ratios and significance levels are given in Table 3. The (+), (-) and (±) indicate anti-atherogenic, pro-atherogenic and indeterminate, respectively.

In this section we present the results for SPA = 0, comparing the case when there is no reversal flow (OSI = 0.0) to the case when there is reversal (OSI = 0.15). In Fig 2 and Table 3 we summarize the results for genes that are significantly altered by reversal flow. Reversal flow significantly altered the expression of 11 genes (p<0.1) (8 genes p<0.05). It reduced the expression of vasoactivity related ENOS and COX2, tight junction protein ZO1, the inflammatory cytokine IL6, and the blood coagulation molecules THBD and EPCR compared to non-reversing flow. Reversal flow also increased the expression of SOD2, PRDX2, VCAM-1, CCL5, and ORL1 compared to non-reversing flow.

**Table 3:** Endothelial genes significantly altered by the condition SPA = 0° R relative to SPA = 0°.

Gene bank	Gene name	Up/Down	SPA 0R / SPA 0	Characteristic	p-value
NM_181037.3	ENOS	Down	0.13	Atheroprotective	0.0111
AF004944	COX2	Down	0.14	Proatherogenic	0.0768
AJ313183.1	ZO1	Down	0.35	Atheroprotective	0.0535
NM_001166522.1	THBD	Down	0.29	Indeterminate	0.0619
BC116074	EPCR	Down	0.09	Atheroprotective	0.0256
NM_174132.2	ORL1	Up	3.50	Proatherogenic	0.0263
BC102064	CCL5	Up	4.50	Proatherogenic	0.0096
NM_173923	IL6	Down	0.09	Proatherogenic	0.0488
NM_201527	SOD2	Up	5.55	Atheroprotective	0.0223
NM_174763.2	PRDX2	Up	2.85	Atheroprotective	0.0864
BC151459.1	VCAM1	Up	9.45	Proatherogenic	0.0144

The third column indicates whether the gene is up- or down-regulated. The fourth column is the ratio of gene expression for the two conditions.

### Effects of OSI for SPA = -180°

**Fig 3:**
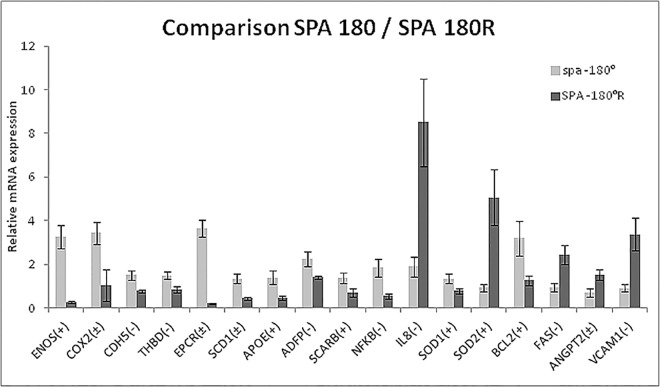
Comparative study of SPA 180 vs SPA 180R. All of the genes that are significantly altered (p<0.1) by reversal flow under the asynchronous condition (SPA 180) are shown in this graph. The ratios and significance levels are given in Table 4. The (+), (-) and (±) indicate anti-atherogenic, pro-atherogenic and indeterminate, respectively.

As shown in Fig 3 and quantified in Table 4, reversal flow altered the expression of 17 genes (p<0.1) (16 genes p<0.05) in the case of asynchronous hemodynamics. Reversal flow reduced the expression of vasoactivity related genes eNOS and COX2, the adhesion molecule CDH5, the coagulation molecules THBD, EPCR and SCD1, the lipid metabolism molecules APOE, ADFP and SCARB, the transcription factor NFkB, the scavenger SOD1 and the apoptosis mediator BCL2—compared to non-reversing flow. Reversal flow significantly increased the mRNA levels of inflammatory molecules IL8 and VCAM1, the reactive oxygen species scavenger SOD2, the death cell receptor FAS and ANGPT2 compared to non-reversing flow.

**Table 4:** Endothelial genes significantly altered by the SPA = -180° relative to SPA = -180° R.

Gene bank	Gene name	Up/Down	SPA 180° R /180°	Characteristic	p-value
NM_181037.3	ENOS	Down	0.08	Atheroprotective	< 0.0001
AF004944	COX2	Down	0.30	Indeterminate	0.0942
XM_002694894	CDH5	Down	0.52	Proatherogenic	0.002
NM_001166522.1	THBD	Down	0.57	Proatherogenic	0.0356
BC116074	EPCR	Down	0.05	Indeterminate	< 0.0001
BC102432	SCD1	Down	0.33	Indeterminate	0.0006
NM_173991.2	APOE	Down	0.33	Atheroprotective	0.0158
BC102211	ADFP	Down	0.63	Proatherogenic	0.028
BC134513	SCARB	Down	0.51	Atheroprotective	0.0503
DQ464067	NFKB	Down	0.29	Proatherogenic	0.0029
EU276073.1	IL8	Up	4.50	Proatherogenic	0.0057
BC102432	SOD1	Down	0.58	Atheroprotective	0.0561
NM_201527	SOD2	Up	5.4	Atheroprotective	0.017
NM_001166486	BCL2	Down	0.39	Atheroprotective	0.0265
NM_174662	FAS	Up	2.63	Proatherogenic	0.0044
NM_001098855.1	ANGTP2	Up	2.17	Proatherogenic	0.0283
BC151459.1	VCAM1	Up	3.73	Proatherogenic	0.0416

### Distribution of NFκB P65

**Fig 4:**
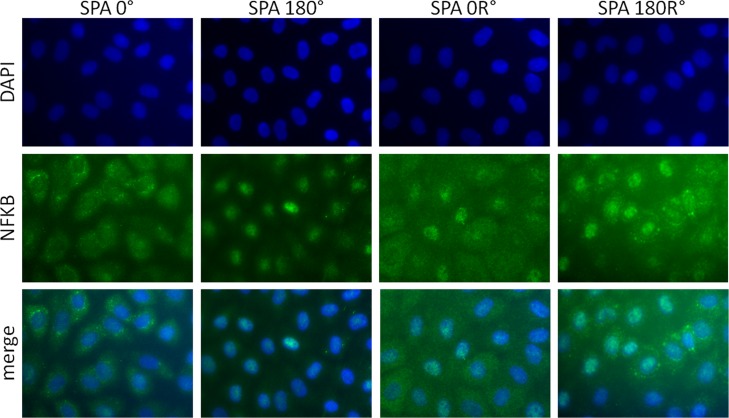
SPA and reversal flow modulate localization and protein expression of NFκB p65. BAEC were exposed to SPA = 0°, SPA = -180°, SPA = 0°R and SPA = -180°R conditions during 7h. Both asynchronous flow and reversal flow promote nuclear translocation of NFκB p65.

We compared the effects of SPA and reversal flow on the protein localization of NFκB p65. We found that SPA = -180° and SPA = -180°R induced the translocation of NFκB p65 to the nucleus as shown in Fig 4. The localization of NFκB p65 was entirely cytoplasmic for SPA = 0° (Fig 4, first column) and a combination of cytoplasmic and partial translocation into the nucleus for SPA = 0°R as shown in Fig 4 (third column).

### Protein Expression of NFκB P65

**Fig 5:**
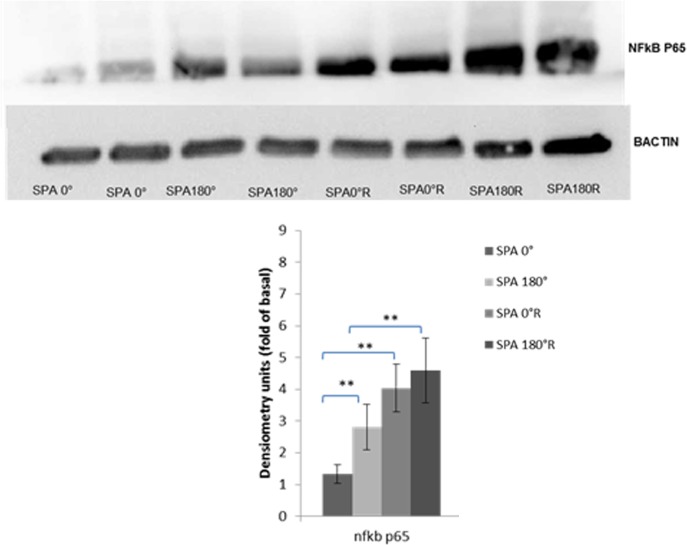
SPA modulates the protein expression of NFkB p65. In the case of synchronous hemodynamics reversal flow enhanced the expression of NFkB p65 compared to non-reversing condition. In the case of asynchronous condition, reversal flow enhanced the protein expression of NFkB p65 compared to the non-reversing condition. Asynchronous hemodynamics with reversal flow modulates protein production of NFκB p65 compared to SPA = 0R. (n = 8 for each case), ** P< 0.05.

To further quantify the effects of SPA and reversal flow on NFκB p65, we compared the protein expression under the four conditions using Western blot analysis as shown in Fig 5. Endothelial cells exposed to asynchronous hemodynamics upregulate NFκB p65 protein production by 2.1 fold (p<0.01) compared to synchronous hemodynamics when there is no reversing flow. In the case of synchronous hemodynamics, reversing-flow upregulates NFκB p65 protein by 3 fold (p<0.001); in the case of asynchronous condition, reversing-flow increases protein production of NFκB by 1.6 fold (p<0.01).

## Discussion

First we examined gene expression of 38 atherosclerosis-related genes under synchronous hemodynamics with reversal flow–denoted SPA = 0°R (OSI = 0.15), and asynchronous hemodynamics with reversal flow–denoted SPA = -180°R (OSI = 0.15) to see if changes in SPA are influential when there is flow reversal. Using a PCR array we determined that BAECS exposed to sinusoidal reversing flow WSS (5±10 dyne/cm^2^) and CS (4 ± 4%) over 7 hours displayed 8 genes that were up regulated by SPA = -180°R relative to SPA = 0°R (Table 2), most of them pro-atherogenic, including NFkB target genes BMP4 and FAS. The upregulation of the transcriptional factor NFκB p65 by SPA = -180°R was determined by western blots and immunofluorescence staining demonstrating the nuclear translocation of NFκB p65 (Figs 4 and 5). Overall our results show that the SPA effect is significant when there is reversing flow. However, in our previous study using the same PCR array and the same values of SPA (0° and -180°), but without reversing flow (OSI = 0), we found that 17 genes were up-regulated by SPA = -180° relative to SPA = 0° (148).

To further understand the interaction of OSI and SPA in endothelial cell responses, we performed a multiple comparisons study between EC exposed to synchronous or asynchronous hemodynamics without reversal (SPA = 0° and SPA -180°) and EC exposed to synchronous or asynchronous hemodynamics with reversal flow (SPA = 0°R, and SPA = -180°R).

The multiple comparisons study between SPA = 0° and SPA = 0°R showed a differential regulation of 11 genes out of 38 (Fig 2) as summarized in Table 3. SPA = 0°R significantly decreased the expression of the vasoactive and atheroprotective genes ENOS and COX2 and the indeterminate genes IL6 and THBD. EC exposed to SPA = 0°R significantly increased the expression of atheroprone genes ORL1 responsible for uptake/binding of oxLDL facilitating plaque formation[13, 14], CCL5 involved in the recruitment of inflammatory cells and proliferation of SMC in the atherosclerotic plaques [15] and VCAM1 involved in leukocyte-endothelial migration and adhesion[16, 17]. SPA = 0°R significantly down regulated the gene expression of EPCR which is critical player in the thrombogenic process and is down regulated in atherosclerotic arteries [18]. SPA = 0° R also upregulated the expression of atheroprotective genes SOD2 which helps minimize the generation of reactive oxygen species [19] and PRDX2 which prevents the formation of fatty streaks and the oxidation of LDL [20]. The overall result is that under the synchronous condition, that is considered to be anti-atherogenic when there is no reversal [10], the introduction of flow reversal induces a pro-atherogenic regulation of 7 genes and an anti-atherogenic regulation of 3 genes (Table 3).

In the comparative case of SPA = -180° and SPA = -180°R. The analysis showed that reversing flow modulates gene expression of 17 of 38 genes (Fig 3 and Table 4). Reversing flow induces a proatherogenic environment with the down regulation of atheroprotective genes ENOS, APOE, SCARB, THBD, BCL2, and SOD1; down regulation of genes with indeterminate function COX2, EPCR, SCD1; but also down regulation of atheroprone genes CDH5, ADFP and NFkB. On the other hand reversing flow up-regulates gene expression of atheroprotective genes SOD2 and IL8, and also upregulates NFKB p65 target genes such as the proatherogenic growth factors ANGPT2 and VCAM1 and the death cell receptor FAS. The overall result is that under the asynchronous condition, that is considered to be atherogenic when there is no reversal [10], the introduction of flow reversal induces a pro-atherogenic regulation of 9 genes and an anti-atherogenic regulation of 5 genes (Table 4).

We examined the protein expression of the major inflammatory transcription factor NFκB p65 under the four different conditions (Fig 5). We observed that SPA = 0°R significantly increased NFκB p65 protein content compared to SPA = 0°, and SPA = -180°R significantly increased NFκB p65 protein content compared to SPA = -180°. But the difference in protein content of NFκB p65 between SPA = 0°R and SPA = -180°R was not significant.

In the study of the intracellular distribution of NFκB p65 (Fig 4), we found that EC subjected to SPA = -180° and SPA = -180°R induced the translocation of NFκB p65 into the nucleus while NFκB p65 is located exclusively in the cytoplasm in SPA = 0°. The case SPA = 0°R also modulates the translocation of NFκB p65 into the nucleus. Reversal flow clearly upregulates NFkB p65 nuclear translocation compared to non-reversal flow for both SPA conditions.

A major observation of the present study is that the SPA = -180° condition always leads to an up-regulation of gene expression relative to the SPA = 0° condition for genes where the differences are significant. This was described previously [10] for the non-reversal case (OSI = 0.0) and in Fig 1 and Table 2 for the reversal case (OSI = 0.15). This may be related to the theoretical prediction that the strain energy of the plasma membrane remains elevated over the entire flow cycle at SPA = -180°, whereas it remains close to zero for nearly half of the cycle when SPA = 0° [21].

Another important observation is that significant changes in gene expression induced by variations in the SPA form 0° to -180° are typically 2–3 fold as shown in [10] for OSI = 0.0, and Table 4 for OSI = 0.15. On the other hand, the significant changes in gene expression induced by changes in the flow pattern from no reversal to reversal are in the range 2–12 fold for SPA = 0° (Table 3), and 2–18 fold for SPA = -180° (Table 4). This suggests that reversal flow can be a stronger stimulus for altered gene expression than asynchronous flow for some genes. There are, however, regions of the circulation where asynchronous flow is dominant and reversal flow is minimal, such as in coronary arteries away from branch points where atherosclerosis often occurs [22, 23]. In these regions, the pro-atherogenic hemodynamic environment is dominated by asynchronous flow, not low shear and reversing flow.

On the outer wall of branch points, where both reversal flow and asynchronous flow (SPA close to -180°) co-localize [8], atherosclerosis is well known to be localized [8, 24]. In such locations asynchronous flow and reversal flow conspire to alter gene expression in a pro-atherogenic direction.

The case of reversal flow without asynchronous flow is not common in the cardiovascular system. In an experimental system in mice that uses partial carotid ligation, it is possible to induce reversing flow in the straight section of the left common carotid artery where the flow is expected to be synchronous [25]. Using this surgical modification in an APOE^-/-^ mouse on a Western diet, rapid plaque development has been observed [25].

In conclusion, both reversal flow and asynchronous flow are important mechanical stimuli altering gene expression in a pro-atherogenic manner in the cardiovascular system. Future experiments should examine the *in vivo* roles of the SPA in vascular biological responses. A study to compare gene expression in vessels characterized by asynchronous hemodynamics such as in the proximal coronary arteries away from branch (asynchronous, no reversal) and the carotid bifurcation outer wall (asynchronous with reversal) and more synchronous hemodynamics such as the common carotid arteries and femoral arteries would be valuable for comparison to the gene expression profiles obtained in the current in vitro studies. The present study has shown the association of asynchronous conditions with the up-regulation and modulation of the NFκB p65 inflammatory pathway. Important future experiments in vitro would explore if the asynchronous condition has the potential to activate other inflammatory pathways such as the important AP1 pathway.
